# Low natural killer (NK) cell counts in peripheral blood adversely affect clinical outcome of patients with follicular lymphoma

**DOI:** 10.1038/bcj.2016.67

**Published:** 2016-08-12

**Authors:** L He, H-Y Zhu, S-C Qin, Y Li, Y Miao, J-H Liang, Y Xia, Y Wang, Y-J Wu, L Wang, L Fan, J-Y Li, W Xu

**Affiliations:** 1 Department of Hematology, the First Affiliated Hospital of Nanjing Medical University, Jiangsu Province Hospital, Nanjing, China; 2 Collaborative Innovation Center for Cancer Personalized Medicine, Nanjing Medical University, Nanjing, China

The Follicular Lymphoma International Prognostic Index (FLIPI) and the Follicular Lymphoma International Prognostic Index-2 (FLIPI-2) have been widely used as models for predicting outcomes in follicular lymphoma (FL) based on clinical parameters.^1, 2^ However, host immunity and tumor microenvironment are not taken into account by either FLIPI or FLIPI-2, which have been demonstrated to remarkably influence the clinical outcomes of patients with FL. Thus, a series of studies have focused on the search for simple and effective surrogate biomarkers that are immunologically relevant and can serve as prognostic factors.

Natural killer (NK) cells are important components of the innate immune response with crucial roles in eliminating viruses, regulating dendritic cells, and killing malignant cells.^3^ NK cell count is a surrogate marker of host immune status. Previously, Plonquet *et al.*^4^ reported that the peripheral blood NK cell count was associated with clinical outcomes of diffuse large B-cell lymphoma patients with age-adjusted International Prognostic Index scores of 2 or 3. To our knowledge, researches regarding prognostic value of peripheral blood NK cell counts in FL are not very well established. Shafer *et al.*^5^ found that low NK cell counts in the blood (0.15 × 10^9^/l) as suggested in earlier reports were associated with inferior OS by univariate analysis (*P*=0.02) and trended toward significance by multivariate analysis (*P*=0.08). To reevaluate the role of NK cell counts in the prognosis of FL, we established this cohort study.

One hundred and thirty-two patients with FL were admitted to the First Affiliated Hospital of Nanjing Medical University, Jiangsu Province Hospital between January 2001 and October 2015, but five of them were lost to follow-up. The diagnostic criteria and clinical management strategies did not change much during the follow-up times. All cases were pathologically confirmed as FL according to 2008 WHO classification. Complete blood cell (CBC) data were collected in the remaining 127 FL patients upon diagnosis following an informed consent. However, only 114 patients' peripheral blood flow cytometry (PBFCM) records at diagnosis for NK cell markers were available. Therefore, we retrospectively reviewed these 114 patients in this study. The counts of peripheral blood NK cells were calculated from the percentages obtained by flow cytometry. NK cells were referred to CD3-CD16+ and/or CD56+ lymphocytes.

Baseline clinical characteristics were totally available, including age, gender, pathological grade, the number of nodal sites involved, bulky lesion, bone marrow involvement, Ann Arbor stage, B symptoms, serum lactate dehydrogenase (LDH) and serum beta-2 microglobulin (β2-MG) (Table 1a). The FLIPI and FLIPI-2 were used for prognostic stratification. High FLIPI scores (high risk) or high FLIPI-2 scores (high risk) were denoted as score ⩾3. Among the patients, 97 (85.1%) cases were treated with rituximab-containing therapy, 9 cases (7.9%) with chemotherapy and 2 cases (1.7%) with radiotherapy. A watch and wait approach was performed at diagnosis for remaining cases (5.3%). CBC and PBFCM analysis indicated that the median NK cell counts at diagnosis were 0.17 × 10^9^/l (range, 0.03 × 10^9^/l−5.08 × 10^9^/l). All *P*-values represented were two-sided, and statistical significance was declared at *P*<0.05. The patients' clinical parameters were analyzed for possible interactions with the level of NK cell counts at diagnosis by using Mann–Whitney *U*-test, but no significant correlation was observed among any groups (Table 1a).

Until December 2015, with a median follow-up of 23 months (range, 1–116 months), the median progression-free survival (PFS) and overall survival (OS) were not reached. The correlation between clinical features and PFS or OS has been analyzed by univariate and multivariate analyses. In this cohort, we analyzed different NK cell counts cutoff points by X-tile. The most discriminative cutoff point was determined to be 0.10 × 10^9^/l for FL because it yielded the greatest difference in PFS and OS. After dichotomization by the optimal cutoff levels, NK cell counts <0.10 × 10^9^/l (low group) predicted for shorter time to progression (*P*=0.001) (Figure 1a) and worse OS (*P*=0.012) in Kaplan–Meier method (Figure 1b). The median PFS was 13.3 months in patients with low NK cell counts group (<0.10 × 10^9^/l), but not reached in those with high NK cell counts group (⩾0.10 × 10^9^/l), whereas the median OS were not reached in both groups. There were five deaths per group (low versus high NK cells), which may be a coincidence owing to either small sample size or relatively short follow-up. The causes of death mainly lay in lymphoma progression, severe infection, hepatic failure, respiratory failure, hemorrhage, pleura effusion, seroperitoneum effusion and extensive involved sites.

As a dichotomised variable, low NK cell counts (<0.10 × 10^9^/l) had an association with inferior PFS and poor OS by univariate Cox regression analysis. The analysis showed that other discrete variables were also related to lower PFS (Table 1b) or shorter OS (Table 1b). Considering FLIPI and FLIPI-2 are widely used prognostic indices of the baseline characteristics of FL, covering proven prognostic factors such as LDH, Hb and β2-MG, only low NK cell counts, FLIPI (high vs low/int.) or FLIPI-2 (high vs low/int.) were entered into the multivariate models, which further revealed that both low NK cell counts (<0.10 × 10^9^/l) and high FLIPI-2 scores (⩾3), as dichotomised variables, maintained their prognostic value for PFS and OS (Table 1c).

As we know, FL cells express high levels of HLA-class I,^6^ which may protect themselves from being recognized by NK cells owing to HLA matching.^5^ Nevertheless, this NK cell-mediated cytotoxicity can be repaired partially by expression of NKG2D ligands on HLA-class I-positive cells. Therefore, there is a possibility that NK cells would have a role in the antitumor efficacy of HLA-class I-positive malignancies including FL,^5^ in accordance with the result of low NK cell levels correlating with inferior outcome in FL.

In this study, NK cell counts were defined as CD3-CD16+ and/or CD56+ lymphocytes. Actually, circulating NK cells can be divided into two main subsets. They are CD56^dim^ and CD56^bright^ cells, respectively. CD56^bright^ cells do not express cytotoxicity markers, but CD56^dim^ cells do.^7^ Besides, peripheral NK cells diversely express functional receptors, combination of which might determine the antitumor ability.^8^ A better knowledge of these various NK cell subsets may help to deepen the understanding of crosstalks between the immune system and follicular lymphoma.

Moreover, rituximab has shown excellent antitumor activity in malignant B-cell lymphomas such as FL. The mechanism of rituximab is currently believed to act through four signaling pathways: antibody-dependent cell-mediated cytotoxicity (ADCC), complement-dependent cytotoxicity, direct signaling triggering apoptosis, and increased sensitivity to chemotherapy.^9^ Most researches exploring ADCC and rituximab have pointed toward interactions of rituximab with CD16 on NK cells.^10^ In this signaling pathway, rituximab recruits NK cells towards malignant B cells via CD16, and the NK cells subsequently eliminate the malignant rituximab-coated cells. It is feasible that CD16 has a role in activating NK cells locally, and that the resulting cytokines produced by NK cells enhances ADCC mediated by other receptors and other cells.^10^ Thus, this privileged mechanism of action of rituximab supports that lower NK cell counts may link to worse outcome with a defected NK cell activity and a decreased rituximab-dependent cellular cytotoxicity.^11^

The results may provide potential of treatment targeting the activation of NK cells, including rituximab, lenalidomide or their combination. Lenalidomide has already shown notable activities in relapsed and refractory FL.^12^ It has a profound effect on NK cells. Through expanding NK cell numbers and enhancing NK cells activity as well as NK-mediated ADCC, the mechanism of lenalidomide action comprises both acquired and innate antitumor immune response.^13^ Moreover, considerable attention has been given to cell therapy. NK-92 cells are reported to have high-selective killing effects against various cancer cells, including myeloma, leukemia, melanoma and breast cancer, in preclinical or clinical setting.^14, 15^ Whether it would have efficacy or not in FL remains unknown.

According to traditional prognostic scoring, FLIPI was significantly associated with shorter survival by univariate analysis, but not maintained the prognostic values in multivariate analysis, which was probably due to the small sample size of the present study, relatively short follow-up or their not reflecting immune systemic mechanisms and the microenvironment.

In conclusion, the baseline peripheral blood NK cell count obtained at diagnosis may represent as an effective biomarker in clinical practice for host immune homeostasis and the tumor microenvironment in FL. Furthermore, this could become the foundation for development of novel therapeutic agents targeting the activation of NK cells.

## Figures and Tables

**Figure 1:**
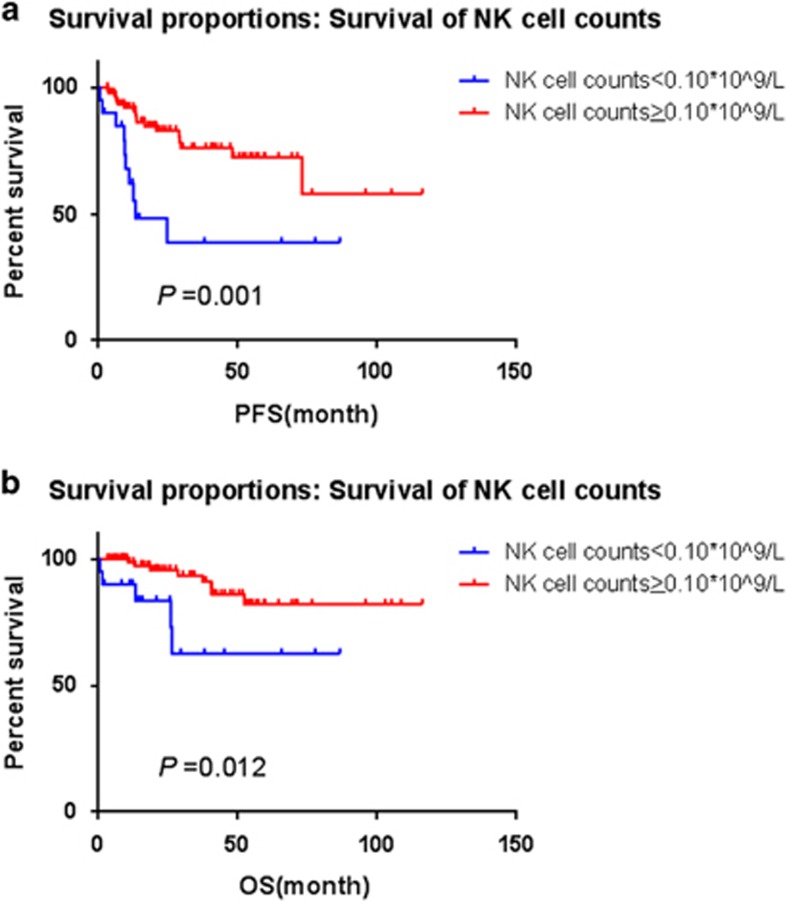
(**a**) Progression-free survival (PFS) of 114 patients with FL according to the absolute natural killer (NK) cell counts at presentation by Kaplan–Meier estimation. (**b**) Overall survival (OS) of 114 patients with FL according to the absolute natural killer (NK) cell counts at presentation by Kaplan–Meier estimation.

**Table 1a:** Clinical characteristics of the 114 FL patients and the differences of ANKC among various groups

*Variables*	*Total (%)*	*Median ANKC (range, × 10*^*9*^*/l)*		P*-value*
*Age*				
⩽60 years	80 (70.9)	0.17	0.03–5.08	0.802
>60 years	34 (29.8)	0.20	0.03–0.63	
				
*Gender*				
Male	64 (56.1)	0.18	0.03–2.22	0.314
Female	50 (43.9)	0.15	0.03–5.08	
				
*Pathological grade*				
1–2	80 (70.2)	0.18	0.03–5.08	0.169
3	34 (29.8)	0.17	0.04–0.63	
				
*Symptoms status*				
A	67 (58.8)	0.18	0.03–5.08	0.413
B	47(41.2)	0.17	0.03–2.22	
				
*Ann Arbor stage*				
I/II	15 (13.2)	0.19	0.05–0.40	0.939
III/IV	99 (86.8)	0.18	0.03–5.08	
				
*Hb*				
⩾120 g/l	71 (62.3)	0.18	0.03–1.34	0.235
<120 g/l	43 (37.7)	0.15	0.03–5.08	
				
*LDH*				
⩽Normal	83 (72.8)	0.17	0.03–2.22	0.693
>Normal	31 (27.2)	0.18	0.05–5.08	
				
*Number of nodal sites*				
⩽4	21 (18.4)	0.26	0.04–0.40	0.402
>4	93 (81.6)	0.17	0.03–5.08	
				
*Bone marrow*				
Uninvolved	64 (56.1)	0.19	0.04–0.96	0.378
Involved	50 (43.9)	0.16	0.03–5.08	
				
*β2-MG*				
⩽Normal	58 (50.9)	0.19	0.04–1.27	0.628
>Normal	56 (49.1)	0.17	0.03–5.08	
				
*Bulky lesion*				
⩽6 cm	95 (83.3)	0.17	0.03–1.27	0.055
>6 cm	19 (16.7)	0.24	0.03–5.08	
				
*FLIPI*				
Low/int. (<3)	57 (50)	0.18	0.04–1.27	0.430
High (⩾3)	57 (50)	0.17	0.03–5.08	
				
*FLIPI-2*				
Low/int. (<3)	78 (68.4)	0.17	0.04–1.27	0.838
High (⩾3)	36 (31.6)	0.18	0.03–5.08	

Abbreviations: ANKC, absolute NK cell counts; β2-MG, beta-2 microglobulin; FL, follicular lymphoma; FLIPI, the Follicular Lymphoma International Prognostic Index; FLIPI-2, the Follicular Lymphoma International Prognostic Index 2; Hb, hemoglobin; LDH, lactate dehydrogenase; Int., Intermediate risk.

**Table 1b:** Univariate Cox regression analysis of the main prognostic factors for PFS and OS in 114 patients with FL

*Prognostic factors*	*Univariate analysis (PFS)*		*Univariate analysis (OS)*	
	*HR (95% CI)*	P*-value*	*HR (95% CI)*	P*-value*
ANKC <0.10 × 10^9^/l	3.492 (1.598–7.634)	**0.002**	3.836 (1.247–11.803)	**0.019**
FLIPI (high vs low/int.)	2.887 (1.226–6.798)	**0.015**	1.846 (0.566–6.015)	0.309
FLIPI-2 (high vs low/int.)	3.293 (1.551–6.994)	**0.002**	3.388 (1.106–10.376)	**0.033**
Age >60 years	2.083 (0.989–4.387)	0.053	1.642 (0.549–4.916)	0.375
Hb <120 g/l	1.692 (0.891–3.284)	0.111	3.556 (1.094–11.561)	**0.035**
LDH>normal	3.463 (1.643–7.297)	**0.001**	3.685 (1.203–11.289)	**0.022**
Stage III/IV	3.689 (0.501–27.168)	0.200	10.275 (0.083–50.495)	0.411
Involved nodal sites >4	1.773 (0.533–5.889)	0.350	0.621 (0.170–2.262)	0.470
β2-MG>normal	3.054 (1.330–7.013)	**0.008**	2.177 (0.670–7.074)	0.196
Bone marrow involvement	2.120 (1.000–4.494)	0.050	2.609 (0.851–7.999)	0.093
Bulky lesion >6 cm	1.181 (0.477–2.923)	0.719	0.755 (0.167–3.414)	0.715

Abbreviations: ANKC, absolute NK cell counts; β2-MG, beta-2 microglobulin; FL, follicular lymphoma; FLIPI, the Follicular Lymphoma International Prognostic Index; FLIPI-2, the Follicular Lymphoma International Prognostic Index 2; Hb, hemoglobin; LDH, lactate dehydrogenase; Int., Intermediate risk; OS, overall survival; PFS, progression-free survival. *P-*value<0.05 in bold font is statistically significant.

**Table 1c:** Multivariate Cox regression analysis of the main prognostic factors for PFS and OS in 114 patients with FL

*Prognostic factors*	*Multivariate analysis (PFS)*		*Multivariate analysis (OS)*	
	*HR (95% CI)*	P*-value*	*HR (95% CI)*	P-*value*
ANKC <0.10 × 10^9^/L	3.497 (1.567–7.800)	**0.002**	3.763 (1.203–11.769)	**0.023**
FLIPI (high vs low/ int.)	1.554 (0.568–4.253)	0.391	/	/
FLIPI-2 (high vs low/ int.)	2.771 (1.135–6.767)	**0.025**	3.318 (1.077–10.224)	**0.037**

Abbreviations: ANKC, absolute NK cell counts; FLIPI, the Follicular Lymphoma International Prognostic Index; FLIPI-2, the Follicular Lymphoma International Prognostic Index 2; Int., Intermediate risk; OS, overall survival; PFS, progression-free survival. *P*-value<0.05 in bold font is statistically significant.
